# Biogeographic differences in plant–soil biota relationships contribute to the exotic range expansion of *Verbascum thapsus*

**DOI:** 10.1002/ece3.6894

**Published:** 2020-10-28

**Authors:** Julia Dieskau, Helge Bruelheide, Jessica Gutknecht, Alexandra Erfmeier

**Affiliations:** ^1^ Institute of Biology/Geobotany and Botanical Garden Martin Luther University Halle‐Wittenberg Halle (Saale) Germany; ^2^ German Centre for Integrative Biodiversity Research (iDiv) Halle–Jena–Leipzig Leipzig Germany; ^3^ Department of Soil, Water, and Climate University of Minnesota Twin Cities MN USA; ^4^ Institute for Ecosystem Research Kiel University Kiel Germany

**Keywords:** common mullein, exotic soil biota exclusion, home‐away comparison, non‐native alien weeds, plant–soil feedback, reciprocal transplant experiment, soil sterilization

## Abstract

1. Exotic plant species can evolve adaptations to environmental conditions in the exotic range. Furthermore, soil biota can foster exotic spread in the absence of negative soil pathogen–plant interactions or because of increased positive soil biota–plant feedbacks in the exotic range. Little is known, however, about the evolutionary dimension of plant–soil biota interactions when comparing native and introduced ranges.
2. To assess the role of soil microbes for rapid evolution in plant invasion, we subjected *Verbascum thapsus*, a species native to Europe, to a reciprocal transplant experiment with soil and seed material originating from Germany (native) and New Zealand (exotic). Soil samples were treated with biocides to distinguish between effects of soil fungi and bacteria. Seedlings from each of five native and exotic populations were transplanted into soil biota communities originating from all populations and subjected to treatments of soil biota reduction: application of (a) fungicide, (b) biocide, (c) a combination of the two, and (d) control.
3. For most of the investigated traits, native populations showed higher performance than exotic populations; there was no effect of soil biota origin. However, plants developed longer leaves and larger rosettes when treated with their respective home soil communities, indicating that native and exotic plant populations differed in their interaction with soil biota origin. The absence of fungi and bacteria resulted in a higher specific root length, suggesting that *V. thapsus* may compensate the absence of mutualistic microbes by increasing its root–soil surface contact.
4. **Synthesis.** Introduced plants can evolve adaptations to soil biota in their new distribution range. This demonstrates the importance of biogeographic differences in plant–soil biota relationships and suggests that future studies addressing evolutionary divergence should account for differential effects of soil biota from the home and exotic range on native and exotic populations of successful plant invaders.

## Graphical abstract

We tested for differential effects of soil microbes from the native and introduced ranges for the exotic range expansion of *Verbascum thapsus* in a reciprocal transplant experiment. We found significant interactions between plant range origin and soil biota origin and suggest that *V. thapsus* may compensate a lack of mutualistic microbes by increasing root surface. Our results emphasize that the evolutionary dimension of plant–soil biota interactions needs to be considered in explaining plant invasions.

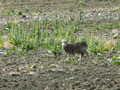

## 1 INTRODUCTION

Exotic plant populations can evolve adaptations to new environmental conditions (Bossdorf et al., 2005), and there is evidence that both abiotic (Hock et al., 2019; Maron et al., 2004) and biotic factors (Gundale et al., 2014; Rogers & Siemann, 2004; Stastny et al., 2005) may foster evolutionary processes in range expansions (Erfmeier, 2013). The role of soil biota in plant invasions has gained substantial attention during recent decades as documented in a significant increase in the number of studies testing the effect of soil biota from the home and/or the exotic range on exotic species (Beckstead & Parker, 2003; Callaway et al., 2011; Maron et al., 2014; Reinhart et al., 2003). Because of their impact on a large number of vital ecosystem processes, plant–soil feedback reactions play a key role in ecosystem functioning (van der Heijden et al., 2008). Interactions between plants and soil microbes, for example, explain a significant proportion of the variance in the relative abundance of species in plant communities (Klironomos, 2002). Although symbiotic associations between vascular plants and mycorrhizal fungi (Klironomos, 2002; Vogelsang et al., 2006), nitrogen‐fixing bacteria (Hayat et al., 2010), or others can significantly increase individual plant productivity and therefore provide a competitive advantage to the host, soil pathogen‐ and root herbivore‐mediated feedbacks can have a strong negative effect on plant performance (Bever, 2003; Callaway et al., 2004) and survival (Bell et al., 2006; Packer & Clay, 2000). In natural systems, positive and negative effects of soil biota can occur simultaneously and may have either a net antagonistic or a synergistic effect or simply offset each other as a null outcome (Reinhart & Callaway, 2006). The net effect on plant establishment, growth, and fertility, however, strongly varies among different species depending on soil biota composition (Agrawal et al., 2005; van der Putten et al., 1993).

One of the main assumptions addressing the role of soil‐borne microorganisms in plant invasions is that the net effect of soil biota on native plant species is negative, whereas its effect on exotic species is positive or neutral, thereby providing an advantage to the colonizer (Callaway et al., 2004; Klironomos, 2002). These differences might be ascribed to a release from belowground enemies during plant establishment in a new distribution area (enemy release hypothesis, ERH) and have been tested with a focus on aboveground enemies such as leaf and seed herbivores (Adams et al., 2009; Joshi & Vrieling, 2005). An alternative explanation offers a positive effect of soil biota from the exotic range compared with the effect of soil biota from the native range, referred to as the enhanced mutualism hypothesis (EMH; Reinhart & Callaway, 2006). Several studies have provided evidence that a lack of mutualists in the new range may impede invasions, however, which can be overcome and supported, in contrast, by the introduction and “coinvasion” of mycorrhizal fungi into new habitats (Diez et al., 2010; Nuñez et al., 2009; Wandrag et al., 2013; Zenni et al., 2017). Hence, the effect of soil biota on plant performance strongly varies and depends on whether the soil biota originates from the native or the exotic range. Given such differences, van der Putten (2014) strongly recommends that studying soil biota in plant invasions requires information from both ranges. Apart from ecological release effects, plant invasions may reflect evolutionary variation in plant–soil interactions (Schweitzer et al., 2014; Yang et al., 2013). Blossey and Notzold (1995) hypothesized that a release from natural enemies will induce a reallocation of resources from defense mechanisms to competitive abilities, which corresponds to the evolution of increased competitive ability (EICA) hypothesis. The belowground dimension of this hypothesis implies that such a release from belowground enemies may induce shifts in root‐associated traits that in turn will translate into invasive exotic plant success (Dawson & Schrama, 2016). Furthermore, as shown in several plant–soil feedback experiments, plants and soil‐borne fungi and bacteria can adapt to each other (Callaway, et al., 2004; Klironomos, 2002; Reinhart & Callaway, 2006). Evolutionary consequences in plant–soil feedbacks can even imply better‐performing plants that translate into fitness advantages when grown in their home soil communities as has been shown for *Trifolium pratense* (Wagg et al., 2015). Experimental approaches addressing range effects of soil biota from native and exotic ranges as plant provenance trials are an appropriate tool to study such evolutionary variation in plant–soil feedback interactions. However, effects of soil‐borne microbes from home and exotic ranges on both native and exotic populations of a successful plant invader have been investigated only in a very limited number of studies and with inconsistent outcome to date (Gundale et al., 2014; Lankau & Keymer, 2018; Shelby et al., 2016).

Our study seeks to (a) understand the role of plant and soil biota origin for the invasion success of *Verbascum thapsus* and (b) identify whether plant–soil feedbacks differ in the native versus. the exotic range. Here, we test for the influence of soil biota from the native and exotic range on native and exotic populations of *V. thapsus* in a reciprocal transplant experiment. We compared the effects of soil‐borne microorganisms from the native (Germany) and the exotic range (New Zealand) each on native and exotic plant origins. To test whether pathogenic or mycorrhizal fungi play a specific role in the invasion of *V. thapsus*, we distinguished between the effects of soil fungi and bacteria by adding differentially selective biocides. We hypothesized that (a) native and exotic *V. thapsus* populations provide evidence for genotypic divergence with exotic populations showing a higher overall plant performance in consequence of a release from natural enemies and a redistribution of resources. Furthermore, we assume that (b) exotic soil biota communities favor plant growth because of a more positive net effect of soil microorganisms from the exotic range compared with soil biota from the native range. As a result, we expect (c) plant–soil feedbacks to differ between ranges as revealed by significant interactions between plant origin and soil biota origin, expressing home‐away effects and thereby indicating the result of adaptive processes.

## 2 METHODS

### 2.1 Study species

*Verbascum thapsus* L. (common mullein, *Scrophulariaceae*) is a monocarpic and self‐fertile herbaceous plant with a native distribution range in Europe and Central Asia from the boreal zone to the Mediterranean area (Ansari & Daehler, 2000; Jäger, 2017). Currently, *V. thapsus* is a common species globally (Ansari & Daehler, 2000), largely naturalized in North America and Australia, for example, present in all states of the United States and considered invasive in some areas of California (Pitcairn, 2000) and in Hawaii (Starr et al., 2003). In New Zealand, the species is listed in the highest category of the New Zealand Naturalised Vascular Plant Checklist as fully naturalized. Common mullein is well adapted to drought stress and typically grows in open anthropogenic sites such as dry, sandy, and calcareous quarries, in ruderal habitats, in gravel beds, along roadsides, and on abandoned lands (Jäger, 2017). The species has a tap root that is associated with arbuscular mycorrhizal fungi (Harley & Harley, 1987). As shown by Francis and Read (1995), mycorrhizal fungi can also reduce the relative growth rate of *V. thapsus* by 25% and the survival rate by 33%; thus, AMF does not necessarily promote plant growth. *Verbascum thapsus* produces up to 180,000 seeds per individual creating a huge persistent seedbank (Darlington & Steinbauer, 1961; Gross, 1980; Kivilaan & Bandurski, 1981). Because of its structural and chemical defense mechanisms, particularly owing to extensive hairiness, the plant is not subjected to grazing but regularly predated by the weevil *Gymnetron tetrum* in the native range (Reinartz, 1984).

### 2.2 Sampling design

Seed material and soil samples from five *V. thapsus* populations each were sampled in January and April 2014 in the exotic (New Zealand = NZ) and native (Germany) range, respectively. Populations were selected out of a pool of 17 and eight populations (in NZ and Germany, respectively) with a minimum distance of 5 km between populations. Populations from sites with extremely low or high soil pH were excluded in order to sample populations from comparable abiotic/edaphic site conditions. Populations chosen for the study experienced a mean annual temperature from 7.3 to 9.2°C (in Germany) and 8.7 to 10.3°C (in NZ), while having an annual average precipitation ranging from 485 to 708 mm in Germany and 503 to 2,208 mm in New Zealand (Table 1). Seed material was kept dry at 4°C until the beginning of the experiment. Within each population, five topsoil samples were randomly taken in the upper 10 cm of the mineral horizon and subsequently pooled by population to more encompass the heterogeneity of the site (Gundale et al., 2019). Soil samples were collected during the hemispheres' respective vegetation period, that is, in January 2014 in New Zealand and in May 2014 in Germany. Subsequent to sampling, all soils were transported in cool boxes and immediately stored at −80°C in the laboratories at Lincoln University (NZ) and Martin Luther University Halle (Germany), respectively. Samples from New Zealand were transported to Germany in April 2014 by maintaining the cold chain with dry ice and stored together with German samples at −80°C to stop microbial activity. After 3 (NZ) and 6 months (Germany), respectively, soil was gradually thawed. To reduce effects of differences in nutrient availability and soil structure, we used standard soil (see below) inoculated with soil washes from the different origins. Fresh soil from each of the ten populations was used to transfer soil‐borne fungi and bacteria into a solution following Wagg et al. (2011). For this purpose, 100 ml of fresh soil was mixed with 500 ml deionized water and shaken by hand for 10 min. Subsequently, the mixture was filtered through a 500‐μm soil sieve. This procedure was run two times for each soil origin to increase the final amount of extracted solutions. The solutions obtained from this process were divided into four equal parts and subjected to treatments of chlortetracycline (80 µg/L), cycloheximide (80 µg/L), a combination of both (80 µg/L), or that served as a control in order to reduce the total amount of bacteria (B), fungi (F), bacteria and fungi (BF), or none, respectively. To differentiate between possible direct physiological effects of the biocides and the true soil biota‐mediated effect, the biocides were additionally applied to plants in sterilized soil without adding soil biota and compared with a control treatment with sterilized water.

**Table 1:** Locations, soil pH, and annual average precipitation and temperature of the German and New Zealand *Verbascum thapsus* populations referred to in the experiment

Population	Latitude	Longitude	Location	Soil pH	Annual average precipitation [mm]	Annual average temperature [°C]
GE 1	51.74860111°N	11.02888061°E	Thale	6.08	708	7.3
GE 2	51.67611306°N	11.77403991°E	Könnern	6.16	485	9.1
GE 3	51.51457161°N	12.06928611°E	Halle Peißen	6.40	490	9.0
GE 4	51.85901954°N	12.23637571°E	Wallwitzburg	6.21	501	9.2
GE 5	51.80837263°N	12.22327977°E	Dessau Kreuzung	5.89	499	9.2
NZ 1	43.83919422°S	170.10975080°E	Mount Cook	4.27	2,208	8.7
NZ 2	44.59018738°S	170.18691928°E	Lake Aviemore	4.36	503	10.3
NZ 3	43.90378389°S	170.12599946°E	Lake Pukaki nord	4.26	1,260	9.6
NZ 4	43.99663429°S	170.46204068°E	Lake Tekapo	4.33	667	8.7
NZ 5	44.07988403°S	170.97951920°E	Opuha River	4.39	909	9.6

Note

Soil pH was measured following Blakemore (1987). Climate data were extracted from WorldClim—Global Climate Data for a time interval of 30 years (from 1960 to 1990).

Abbreviations: GE, Germany; NZ, New Zealand.

### 2.3 Greenhouse study

The experiment took place in autumn 2014 in the greenhouse cabins of the Botanical Garden at Martin Luther University Halle. To prevent a contamination of samples and experimental units by ambient biota, we applied sterilization procedures for the greenhouse cabins (0.3% Wofasteril E400, Kesla Pharma Wolfen GmbH, Germany), the seeds (rinsing 15 min 0.3% sodium hypochlorite solution), and the sand and soil used for seedling cultivation and growth (24 hr at 180°C, 8 hr break, 24 hr at 180°C). *Verbascum thapsus* seedlings grew on pure sand to facilitate later transplantation and were fertilized every 10 days with 0.4% Wuxal Universal (Hauert Manna Düngerwerke GmbH, Nürnberg, Germany). After 10 weeks, plants were transplanted into pots with 2 L of the sterilized soil/sand mixture. Pots were thereafter randomly assigned to biocide treatments and inoculated with the respective soil washes (10 ml microbial solution/1 L soil). Each ten plants of the ten origins were grown in all soil biota communities in a reciprocal design, that is, originating from all populations and each subjected to all treatments of soil biota reduction, yielding a total number of 400 experimental units (i.e., individuals). Because of their fast growth during the experiment, plants were repositioned anew in two cabins after 4 weeks. Thereafter, all plants from population NZ2, NZ4, GE1, GE3, and GE5 were randomly allocated to cabin 1, and all other plants remaining from populations NZ1, NZ3, NZ5, GE2, and GE4 were newly arranged in cabin 2. Pots were randomly positioned on benches, and saucers were used to avoid microbial cross‐contamination from neighboring pots. During the experimental period of 12 weeks, plants were watered every second day with deionized water, fertilized every 21 days with 0.4% Wuxal Universal and subjected to additional illumination from 5 a.m. to 9 a.m. and 5 p.m. to 9 p.m. to ensure long‐day exposure to light according to a 16‐hr/8‐hr day/night cycle. The temperatures ranged between 28° and 15°C (day/night), accordingly. Every 3 weeks, rosette area size, leaf number, and leaf length of the largest fully developed nonsenescent leaf were monitored. For the calculation of leaf life span, leaves were marked after 6 and 9 weeks to distinguish between old leaves that already existed at the previous monitoring date, the newly emerged leaves, and the total leaf number at the initial date. By this, we were able to separate losses of previously present leaves from true increases because of emergences of new leaves (King, 1994). Leaf life span was calculated by dividing the number of leaves per plant by the average of leaf production and leaf loss rates. After 12 weeks, plants were monitored for survival and the largest fully developed nonsenescent leaf was harvested to determine the leaf fresh weight, area, length, width and dry weight per individual (after 48 hr at 60°C). This information was used for the calculation of specific leaf area (SLA) and leaf dry matter content (LDMC). Total carbon and nitrogen on the leaf sample level were determined with a nitrogen analyzer (vario EL cube, Elementar Analysensysteme GmbH) and used for C:N ratio calculation. Above‐ and belowground biomass was harvested separately. For the assessment of specific root length (SRL), roots were rinsed with water, and root area and length were measured suspended in water with a transmitted light scanner (Epson Expression 10000 XL, software package WinRHIZO). Both roots and shoots were dried for 72 hr at 80°C and weighed to obtain dry biomass by fraction and to calculate total biomass and root–shoot ratio (RSR).

### 2.4 Statistical analysis

Continuously distributed response variables were analyzed with a linear mixed model (using the package lmerTest in R, version 3.4.0., R Core Team, 2017; Kuznetsova et al., 2017). Residual plots of each selected model were examined to ensure random distribution of model residuals. Accordingly, response variables SLA, RSR, SRL (log), and belowground biomass (sqrt) were transformed as recommended by Zuur et al. (2009) to obtain normal distribution of residuals. Plant origin (PO; with levels: native (German) versus. exotic (New Zealand) population origin) and soil biota origin (SO; with levels: German versus. New Zealand soil origin) and soil biota treatment (T; with levels: C, B, F, BF) were considered fixed factors and tested for all interactions. Soil biota population was considered random and nested in soil biota origin, and plant population as a random factor was nested in plant origin and the greenhouse cabin. Cabin identity was considered an additional random factor in all models. We calculated *F* and *p* values using a type III ANOVA and Satterthwaite's method to calculate the degrees of freedom. Soil biota effects were subjected to Tukey's post hoc tests in order to identify significant differences among treatments. For analysis of repeatedly monitored variables, monitoring date was used as an additional covariate of time and the individual plant identity (ID) nested in cabin identity as an additional random factor. A model simplification via backward selection and comparison of the Akaike's information criterion (AIC) resulted in better/smaller values for the simplified models. Since, by general trend, there was no substantial difference between the models' outcomes, we used the original complete models for reasons of comparability. Survival data were analyzed using a generalized linear mixed effect model (glmer) with the same random and fixed factors as described above. *p* values were calculated using the degrees of freedom from the lmer model.

## 3 RESULTS

### 3.1 Effects of plant origin and soil biota origin

**Figure 1:**
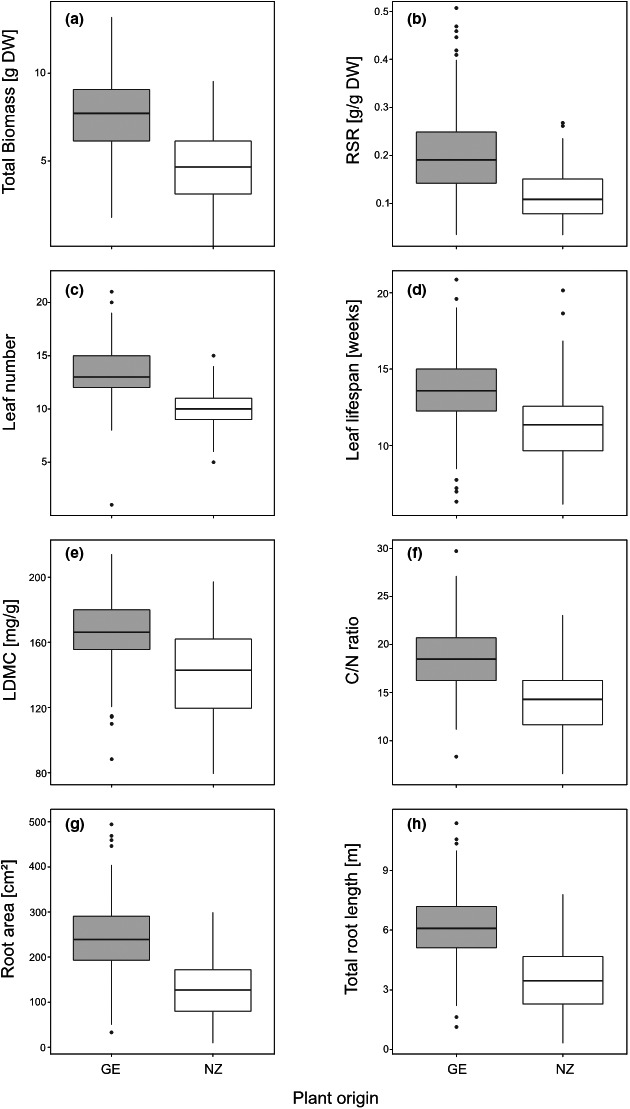
Significant origin effects for (a) total biomass, (b) root–shoot ratio (RSR), (c) leaf number, (d) leaf life span, (e) LDMC (leaf dry matter content), (f) CN ratio, (g) root area, and (h) total root length of native German (GE; gray boxes) and exotic New Zealand (NZ; white boxes) plants. For statistical details, see Table 2

The analysis of survival rate, productivity, leaf, and root traits revealed that plant performance revealed significant differences in many of these variables depending on the populations' origin (Table 2; Figure 1). At the end of the experiment, 91% of native German plants and 68.5% of exotic New Zealand plants had survived (*F*_1,8.00_ = 10.94, *p* = 0.011). Further differences between plant origins were mainly found for productivity and root traits with native German origins displaying 36% higher total biomass (Figure 1a; *F*_1,8.11_ = 33.17, *p* < 0.001) with both larger aboveground (*F*_1,7.99_ = 31.10, *p* < 0.001) and belowground biomass (F_1,8.18_ = 17.26, *p* = 0.003) and a wider root–shoot ratio (Figure 1b; *F*_1,8.16_ = 9.55, *p* = 0.015). In addition, native German plants also had a significantly higher leaf number (Figure 1c; *F*_1,8.15_ = 17.86, *p* = 0.003), associated with an increased leaf life span (Figure 1d; *F*_1,7.56_ = 9.29, *p* = 0.017), a higher LDMC (Figure 1e; *F*_1,8.22_ = 19.41, *p* = 0.002), a higher C‐content (*F*_1,7.92_ = 13.73, *p* = 0.006), and a larger C:N ratio (Figure 1f; *F*_1,8.19_ = 22.80, *p* < 0.001), while N‐content (*F*_1,8.17_ = 23.32, *p* < 0.001) in German origins was lower than in New Zealand plants. There was no such difference in rosette area (*F*_1,7.08_ = 2.11, *p* = 0.189), leaf length (*F*_1,7.35_ = 0.37, *p* = 0.560), leaf width (*F*_1,7.02_ = 0.66, *p* = 0.443), leaf area (*F*_1,7.01_ = 1.30, *p* = 0.292), and SLA (*F*_1,8.12_ = 4.30, *p* = 0.071) between both origins (Table 2). However, native German plants developed roots with increased root area (Figure 1g; *F*_1,8.21_ = 23.52, *p* < 0.001) and total root length (Figure 1h; *F*_1,8.22_ = 27.64, *p* < 0.001) when compared to individuals of exotic New Zealand origin.

**Table 2:** Overview table summarizing the effect of plant origin, soil biota origin, biocide treatment, and the corresponding interaction effects on survival rate and all investigated productivity, leaf, and root traits

	Plant origin				Soil biota origin			Biocide treatment			Plant origin × Soil biota origin			Plant origin × Biocide treatment			Soil biota origin × Biocide treatment			Plant origin × Soil biota origin × Biocide treatment		
	*df*	*F*	*p*	Direction	*df*	*F*	*p*	*df*	*F*	*p*	*df*	*F*	*p*	*df*	*F*	*p*	*df*	*F*	*p*	*df*	*F*	*p*
Survival rate		10.94	**0.011**	GE > NZ		0.66	0.439		0.37	0.772		0.92	0.338		1.23	0.299		0.37	0.772		0.05	0.987
Productivity traits																						
Total biomass	8.11	33.17	**<0.001**	GE > NZ	289.41	0.10	0.755	290.39	0.20	0.897	289.41	0.05	0.833	290.39	0.73	0.535	290.00	0.52	0.669	290.00	0.52	0.668
Aboveground biomass	7.99	31.10	**<0.001**	GE > NZ	289.32	0.17	0.676	290.39	0.22	0.879	289.32	0.08	0.779	290.39	0.70	0.553	289.97	0.47	0.704	289.97	0.62	0.602
Belowground Biomass	8.18	17.26	**0.003**	GE > NZ	8.37	0.02	0.890	289.85	0.42	0.736	289.38	0.03	0.853	288.78	1.10	0.348	289.41	0.68	0.568	288.43	0.57	0.635
Root–shoot ratio	8.16	9.55	**0.015**	GE > NZ	8.12	0.07	0.805	284.04	1.79	0.150	284.29	0.57	0.451	283.45	2.52	0.058	283.88	0.68	0.562	283.32	0.60	0.613
Rosette area	7.08	2.11	0.189		8.07	0.08	0.785	289.04	0.79	0.503	287.43	5.97	**0.015**	287.94	0.66	0.579	287.80	0.63	0.599	286.53	0.98	0.404
Leaf number	8.15	17.86	**0.003**	GE > NZ	294.31	0.11	0.741	294.89	0.32	0.808	294.31	0.08	0.783	294.89	0.73	0.536	294.48	0.52	0.670	294.48	0.38	0.771
Leaf traits																						
Leaf area	7.01	1.30	0.292		8.22	0.20	0.669	285.52	2.90	**0.035**	283.67	0.13	0.718	284.86	1.49	0.216	284.40	0.62	0.605	283.70	1.31	0.271
Leaf length	7.35	0.37	0.560		8.05	0.10	0.761	289.80	2.52	0.058	289.20	5.17	**0.024**	289.04	0.34	0.799	289.32	0.29	0.835	288.60	0.63	0.597
Leaf width	7.02	0.66	0.443		8.20	0.82	0.390	290.96	2.27	0.081	288.84	0.30	0.584	289.97	0.99	0.398	289.30	0.81	0.489	288.35	0.99	0.397
SLA	8.12	4.30	0.071		288.35	0.08	0.784	289.11	0.46	0.709	288.35	1.25	0.265	289.11	0.60	0.618	288.61	2.18	0.090	288.61	1.08	0.357
LDMC	8.22	19.41	**0.002**	GE > NZ	294.52	0.24	0.626	295.56	0.46	0.707	294.52	0.41	0.521	295.56	0.94	0.419	294.84	1.30	0.275	294.84	0.81	0.487
C‐content	7.92	13.73	**0.006**	GE > NZ	7.77	0.44	0.525	289.28	0.72	0.540	287.68	2.03	0.155	288.52	2.36	0.071	287.84	1.10	0.351	287.12	0.91	0.434
N‐content	8.17	23.32	**<0.001**	NZ > GE	293.47	0.15	0.696	294.37	0.90	0.440	293.47	0.11	0.743	294.37	1.22	0.302	293.70	0.90	0.444	293.70	0.90	0.442
CN ratio	8.19	22.80	**<0.001**	GE > NZ	8.21	0.01	0.929	288.71	0.54	0.654	288.17	0.00	0.947	288.01	0.91	0.434	288.08	1.02	0.384	287.43	0.84	0.473
Leaf life span	7.56	9.29	**0.017**	GE > NZ	278.49	0.48	0.489	278.94	1.47	0.222	278.49	0.20	0.652	278.94	0.54	0.654	278.70	0.42	0.741	278.68	0.84	0.474
Root traits																						
Root area	8.21	23.52	**<0.001**	GE > NZ	7.70	0.05	0.830	288.85	2.94	**0.034**	288.44	2.46	0.118	287.78	0.39	0.762	288.36	0.70	0.555	287.33	2.29	0.078
Total root length	8.22	27.64	**<0.001**	GE > NZ	7.74	0.12	0.733	213.70	4.21	**0.006**	213.29	2.80	0.096	212.92	0.10	0.961	213.28	0.81	0.492	212.52	2.92	**0.035**
SRL	8.26	3.76	0.087	GE > NZ	293.62	0.69	0.406	294.95	17.41	**<0.001**	293.62	1.77	0.184	294.95	2.13	0.096	294.04	0.15	0.930	294.04	0.67	0.571

Not<del author="Julia Dieskau" command="Delete" timestamp="1602488999560" title="Deleted by Julia Dieskau on 12.10.2020, 09:49:59" class="reU3">e</del>

Bold numbers indicate significant effects (*p* < 0.05). *df*, degrees of freedom; *F*, *F* value; *p*, *p* value. *F* and *p* values are calculated using a type III ANOVA and Satterthwaite's method.

**Figure 2:**
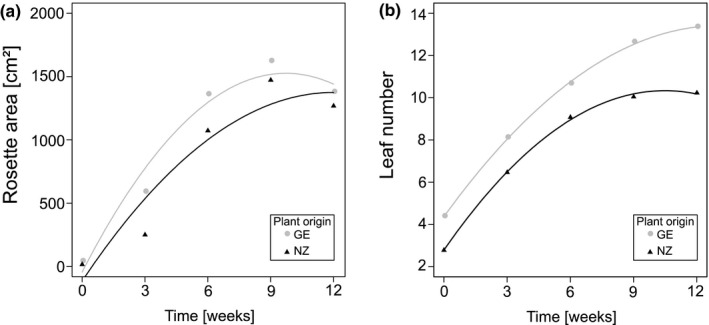
(a) Rosette area and (b) leaf number of *V. thapsus* plants from Germany (native) and New Zealand (exotic) monitored over the experimental period of 12 weeks. For statistical details, see Table 3

For repeatedly monitored variables, time analysis displayed additional significant effects of plant origin and plant origin × time interactions (Table 3). During the first weeks of the experiment, native German plants had longer leaves (F_1,10.39_ = 27.40, *p* < 0.001) and a larger rosette (Figure 2a; *F*_1,10.50_ = 7.30, *p* = 0.021) compared with exotic populations from New Zealand. For both origins, leaf length and rosette area more than tripled in size with a maximum after 9‐week growth. All plants had their maximum leaf number at the end of the experiment, but plants from New Zealand displayed a lower increase in leaf number after the second monitoring date at 6 weeks (Figure 2b; *F*_1,1,363.39_ = 17.37, *p* < 0.001).

**Table 3:** Results of the mixed model analysis for Rosette area, leaf length, and leaf number with the time

	Rosette area			Leaf length			Leaf number		
	*df*	*F*	*p*	*df*	*F*	*p*	*df*	*F*	*p*
PO	10.50	7.30	**0.021**	10.39	27.40	**<0.001**	9.45	10.83	**0.009**
SO	1,444.30	0.10	0.812	1623.07	0.70	0.398	1,114.44	0.66	0.415
B	1,444.00	0.10	0.943	1623.31	0.10	0.939	1,114.37	0.10	0.961
T	1,407.10	3,217.60	**<0.001**	1623.40	4,635.40	**<0.001**	1,363.39	2,995.74	**<0.001**
SO** **×** **PO	1,444.30	0.10	0.790	1623.07	0.00	1.000	1,114.44	0.51	0.477
PO** **×** **B	1,444.00	0.10	0.972	1623.31	0.40	0.729	1,114.37	0.85	0.469
SO** **×** **B	1,444.30	0.20	0.882	1623.13	0.40	0.736	1,114.50	0.28	0.836
PO** **×** **T	1,407.10	0.00	0.895	1623.40	52.80	**<0.001**	1,363.39	17.37	**<0.001**
SO** **×** **T	1,405.90	0.00	0.992	1623.17	0.20	0.668	1,362.51	0.09	0.763
B** **×** **T	1,406.60	0.30	0.841	1623.33	0.60	0.610	1,362.97	0.44	0.724
PO** **×** **SO ×** **B	1,444.30	0.20	0.926	1623.13	0.60	0.645	1,114.50	0.33	0.805
PO** **×** **SO × T	1,405.90	1.60	0.213	1623.17	0.30	0.607	1,362.51	0.08	0.774
PO** **×** **B ×** **T	1,406.60	0.30	0.829	1623.33	0.10	0.954	1,362.97	1.33	0.264
SO** **×** **B ×** **T	1,406.10	0.10	0.941	1623.22	0.20	0.903	1,362.65	0.40	0.755
PO** **×** **SO ×** **B** **×** **T	1,406.10	0.50	0.708	1623.22	0.30	0.839	1,362.65	0.40	0.750

Note

Degrees of freedom (*df*), *F*‐statistics (*F*), and significance (*p*) values are provided. PO = plant origin, SO = soil biota origin, B = biocide treatment, T = time and the corresponding interaction. Bold numbers indicate significant effects (*p* < 0.05). *F* and *p* values are calculated using a type III ANOVA and Satterthwaite's method.

**Figure 3:**
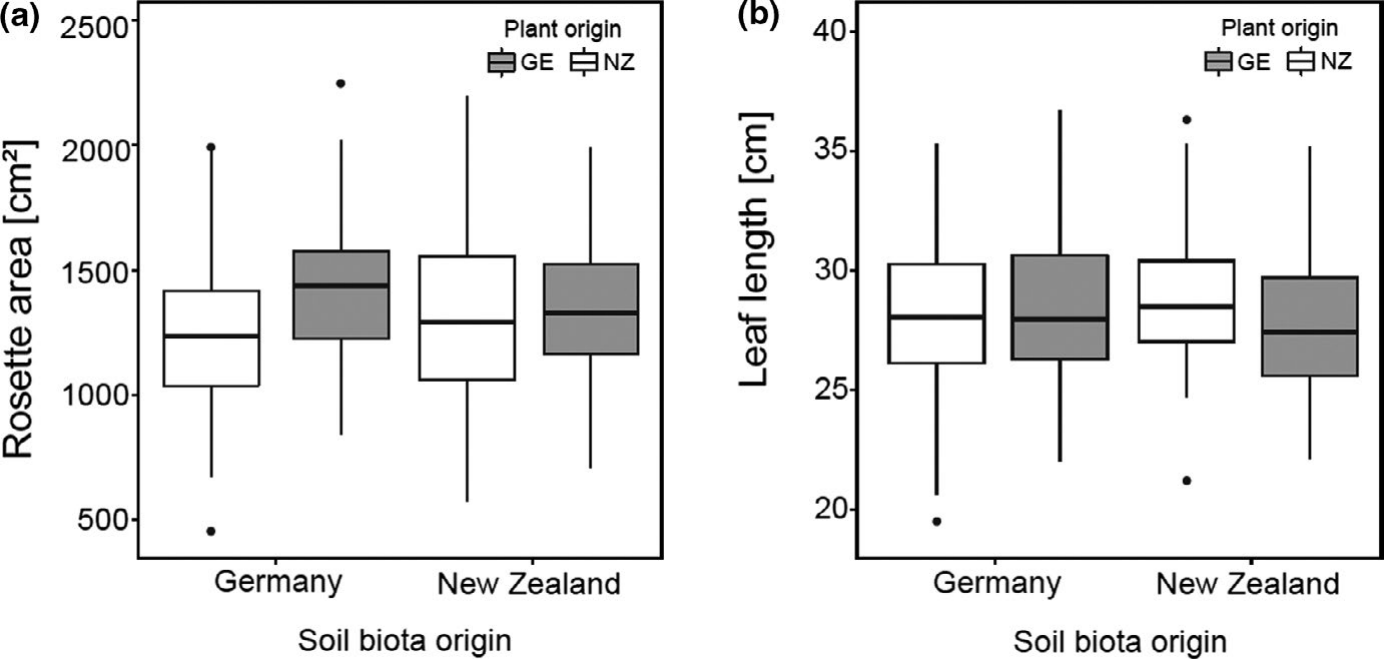
Effects of soil biota from Germany and from New Zealand on (a) Rosette area and (b) leaf length of *V. thapsus* from Germany (native; gray boxes) and New Zealand (exotic; white boxes). For statistical details, see Table 2

The soil biota origin failed to display any main effect on the investigated plant traits; that is, soil biota communities from native and exotic plant ranges did not differ in their effects on plant performance. However, there was a significant plant origin × soil biota origin interaction effect for rosette area (*F*_1,287.43_ = 5.97, *p* = 0.015) and leaf length (*F*_1,289.20_ = 5.17, *p* = 0.024), indicating differences between native and exotic populations in response to soil biota origins. For exotic NZ plant origins, responses in leaf length and rosette area were similar when grown in German or in NZ soil biota communities. In contrast, responses in native German plant origins decreased in magnitude when grown in NZ soil biota communities if compared to treatments with German soil biota, thereby indicating a significant home‐away effect (Figure 3).

### 3.2 Effects of biocide treatment

**Figure 4:**
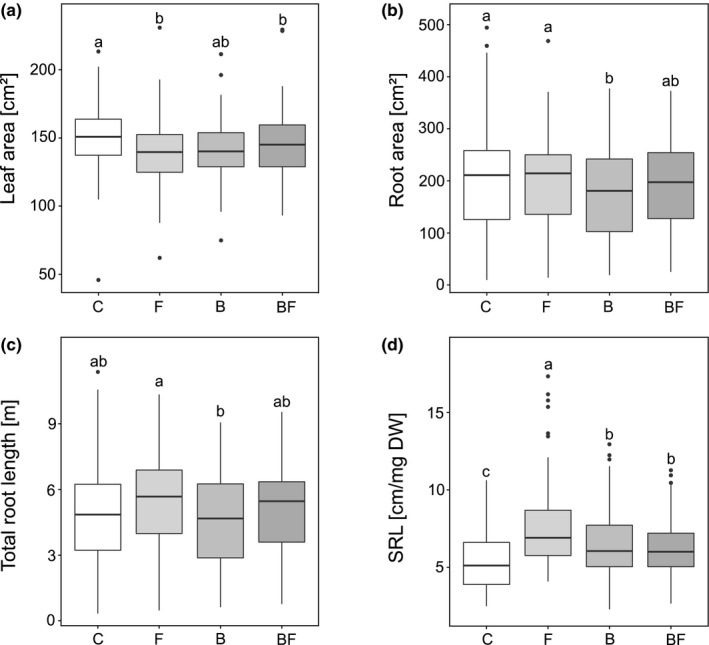
Treatment effects for (a) leaf area, (b) root area, c) total root length, and d) SRL (specific root length) of *V. thapsus* growing with biocide applications: C = control (i.e., no biota exclusion); B = bactericide (i.e., exclusion of bacteria); F = fungicide (i.e., exclusion of fungi); BF = bactericide/fungicide combined (i.e., exclusion of bacteria and fungi). Small letters on top of the boxes indicate significant differences according to the Tukey post hoc analysis. For statistical details, see Table 2

The reduction in different soil biota significantly affected leaf area (Table 2; Figure 4a; *F*_3,285.52_ = 2.90, *p* = 0.035), and all investigated root traits including root area (Figure 4b; *F*_3,288.85_ = 2.94, *p* = 0.034), total root length (Figure 4c; *F*_3,213.70_ = 4.21, *p* = 0.006), and specific root length (Figure 4d; *F*_3,294.95_ = 17.41, *p* < 0.001), but there was no effect on any of the other analyzed productivity variables and leaf traits (Table 2). Leaf area was significantly decreased in treatments where fungicide application was involved; however, leaf area responses to bacteria reduction did not differ significantly from the control treatment (Figure 4a). Root area was reduced when bacteria were excluded, whereas an exclusion of fungi or both did not significantly affect the root area. The total root length was not affected by the biocide treatments when compared to the control; however, plants growing in the fungi exclusion developed significantly longer roots than plants growing in the bacteria exclusion (Figure 4c). The application of biocides always induced a significant increase in SRL if compared to the control. This effect was strongest when fungi were exclusively reduced and still significantly different from bactericide‐only and combined bactericide/fungicide treatments (Figure 4d).

## 4 DISCUSSION

We found consistent evidence for genetic divergence in *V. thapsus* populations from the home versus the exotic range. However, for most traits studied, native populations outperformed the exotic populations. Against our expectation, we did not find effects of soil biota origin on plant performance, but there was evidence provided that native and exotic plant populations differed significantly in their interaction with soil biota from the native and exotic range.

### 4.1 Plant origin effects—native and exotic populations differ strongly

The strong origin effects with native *V. thapsus* populations displaying better performance than plants from the exotic range supported the hypothesis of genetic divergence between native and exotic population: The direction of the differences, however, contradicted our expectation of increased performance in exotic plant material. Plants of native German origin produced higher below‐ and aboveground biomass and invested in a faster development of a large rosette. Although experimental studies on a multiple species basis have often indicated higher performance of exotic origins (Beckmann et al., 2014; Blumenthal & Hufbauer, 2007; Colautti et al., 2009), there are nevertheless contradictory findings showing that increased growth of exotic plant origins as, for example, predicted by the EICA hypothesis (Blossey & Notzold, 1995), is not universal (Parker et al., 2013). More specifically, for *V. thapsus*, plant performance has been studied along environmental gradients in native and non‐native ranges and was found not to consistently differ between ranges (Seipel et al., 2015). In contrast, field studies and experimental common garden studies revealed better performance of introduced populations in the United States when compared to native European populations (Alba et al., 2011; Alba & Hufbauer, 2012; Endriss et al., 2018). In the present study, the pattern was completely reversed and there are several scenarios that might explain this outcome. First, it is possible that the differences encountered may be partially due to covarying effects of different abiotic conditions in native and exotic ranges. Site conditions of populations investigated in this study did not only differ in annual average temperature and precipitation (Table 1) but also varied significantly in soil pH with much lower values for populations from New Zealand (between 4.68 and 5.75) compared with those from Germany (between 6.94 and 7.67; Table 1), therefore presumably also varying in resource supply. Alba and Hufbauer (2012) was able to identify reduced performance of exotic *V. thapsus* populations compared with native ones, when accounting for additional abiotic differences among ranges. In contrast to our results, a common garden experiment in the United States revealed exotic *V. thapsus* populations from the United States to have significantly larger shoot biomass than native European ones (Alba et al., 2011). However, our study system involved exotic NZ–native EU comparisons where levels of abiotic resources displayed in the introduced and native range may be different from exotic US–native EU comparisons. These contradictory results point to the importance of the exact range identity and associated environmental conditions when comparing native and exotic ranges studied (Endriss et al., 2018). This highlights the need to expand biogeographic comparisons of exotic species to multiple region studies involving exotic ranges with different levels of resource supply to quantify the impact of qualitative and quantitative abiotic changes and their contribution to evolutionary divergence (Erfmeier, 2013). Second, apart from effects of abiotic differences among regions, experienced differences in biotic conditions, such as in competition, may have evoked divergence in plant origin responses. Increased size as indicated by rosette growth superiority of native German plants has the potential to limit the growth of competitors and is thus often advantageous in highly competitive environments. This matches with our field observations of higher productivity of co‐occurring species in German populations when compared to neighboring plant species in New Zealand populations. Alba and Hufbauer (2012) precisely elaborated that density of *V. thapsus* was negatively related to vegetation cover. In addition, there is experimental evidence that increased growth and performance in exotic origins if compared to native ones may only become evident under situations of competition, as was shown specifically for *V. thapsus* (Kumschick et al., 2013) and in a meta‐analysis including 27 studies (Felker‐Quinn et al., 2013). Increased performance in exotic origins may only pay off when there is competition with neighboring (native) plants and thus not be evidenced in isolated settings as done in the present study. A third explanation for contradictory outcomes in different exotic ranges is that patterns of genetic diversity and associated effects might depend on particular histories of introductions into regions. *Verbascum thapsus* was most probably introduced to New Zealand in the mid‐nineteenth century with the second immigration wave (Esler, 1987). It is conceivable that a limited number of founder populations functioned as a source for its spread throughout the island resulting in a reduced genetic diversity (Austerlitz et al., 2000; Dlugosch & Parker, 2008; Schrieber & Lachmuth, 2017) and, as a consequence, reduced plant performance in the new distribution area (Ellstrand & Elam, 1993; Keller & Waller, 2002). As founder effects are governed by chance events, the extent of genetic bottlenecks might strongly differ among different regions of introduction (Lachmuth et al., 2010).

Although we cannot address the role of differences in genetic diversity here, we may suppose that the observed patterns possibly could be ascribed to evolutionary processes that have taken place as a consequence of differences in abiotic environmental factors and competitive pressure in the native and exotic range.

### 4.2 Soil biota—no release from enemies and enhanced mutualism in the exotic range

The unexpected lack of differences in the effect of soil biota from the native and the exotic range on the analyzed traits suggests that there is neither evidence for a release from belowground microbial enemies nor for an enhanced mutualism in the exotic range of *V. thapsus*. This contradicts the results of other studies that support the hypothesis of a release from enemies and/or enhanced mutualism in the exotic range as was, for example, shown for several tree (Gundale et al., 2014; Reinhart & Callaway, 2006; Yang et al., 2013) and forb species (Maron et al., 2014; McGinn et al., 2018; Parepa et al., 2013). In contrast, other studies revealed negative feedbacks in exotic forb expansion, for example, of *Centaurea solstitialis* (Andonian et al., 2011). Apparently, there seem to be some differential effects on plant–soil feedbacks depending on the life‐form. In their meta‐analysis, Meisner et al. (2014) concluded that plant–soil feedbacks based on the provenances' own soils could bring about the full suite of positive, negative, and neutral effects, with native plants rather displaying negative and exotics showing positive feedbacks. In addition, origins differed in their responses by life‐form with exotic forbs displaying positive effects and native forbs rather showing negative plant–soil feedbacks, whereas for trees, this trend was reversed. An absence of soil biota origin effects as found in our study was also shown by Beckstead and Parker (2003) for the European beach grass *Ammophila arenaria*. In their study, seedlings were grown in two greenhouse experiments in the home and exotic range. Comparisons with settings in sterilized soil revealed that their performance was reduced both in the native and in the exotic range, thus failing to find a release from natural enemies. Hence, the absence of effects of soil biota origin on overall plant performance is not uncommon.

There are several possible explanations that could underlie our findings. First, soil microbe identities and composition in German and New Zealand *V. thapsus* populations might not have differed significantly from each other. However, given that microbes are not exempt from the fundamental evolutionary processes of geographic isolation and natural selection (reviewed in Rout & Callaway, 2012), this scenario is rather unlikely. A second scenario might be that soil microbes from the two origins, in fact, differ in their identity but not in their overall net effect on traits measured in this study. The absence of soil biota origin effects may arise from conflicting patterns when, for example, populations seem to be adapted to whole microbial communities at their home site while being maladapted to particular microbial populations present at the same time and site (Lankau & Keymer, 2018). In that case, a neutral response may simply be the net outcome of outbalanced antagonistic responses. It is important to take into account the possibility that potentially mutualistic soil microbes from the exotic range, dominated by low pH, did not perform optimal in the less acid‐sterilized German soil used for the experiment. Admittedly, one might consider that different biocides may provide weak or weird results in some cases, but certainly not all.

In our study, soil‐borne microbes mainly affected root traits and leaf area of *V. thapsus*. Compared with the control, the exclusion of soil fungi evoked an increase in SRL and a decrease in leaf area. The exclusion of bacteria produced similar but weaker results. The additional experiment in sterilized soil without soil biota revealed no physiological effect of the biocide treatment on the leaf area and the SRL of *V. thapsus* (data not shown). Thus, we infer that the observed patterns were truly caused by the reduction in soil fungi and bacteria and not by the application of biocide. In general, an increased root–soil surface contact per unit of biomass due to high SRL is associated with a greater physiological capacity for nutrient and water uptake (Ostonen et al., 2007; Perez‐Harguindeguy et al., 2013) and less mycorrhizal dependency (Eissenstat, 1992). Accordingly, there is reason to suggest that *V. thapsus* is compensating the absence of mutualistic microbes by a reallocation of resources from leaves in an increased SRL. However, the nonsignificant interaction between biocide treatment and soil biota origin indicates that the positive effect of soil‐borne fungi and bacteria is not crucial for the invasion success of *V. thapsus* in New Zealand. It is important to mention that we only analyzed soil‐borne microorganisms and not the whole soil biota community. However, nematodes, for example, were also shown to globally attack local native plant species significantly stronger than an exotic invader (van der Putten et al., 2005). Admittedly, interpretation of soil biota effects on plant performance to some extent is limited when studying effects of isolated soil biota components (Callaway et al., 2011). In addition, in the present study, we applied an exclusion treatment of soil biota directly on fresh soil samples from the field, whereas many experiments studying plant–soil feedbacks rely on more than one generation in soils, which might also contribute to different outcomes. Therefore, a prominent role of plant–soil feedbacks for explaining the successful exotic range expansion of *V. thapsus* in New Zealand cannot be completely ruled out.

### 4.3 Interaction between plant and soil biota origin

The outcome of our analyses on selected plant leaf traits provides evidence that *V. thapsus* populations and soil microbes from the native and the exotic range have adapted to each other. When treated with their home soil biota communities, plants developed longer leaves and larger rosettes, which provides them with a high competitive strength for the suppression of nearby seedlings (Grime, 2006; Werner, 1977) and is positively correlated with the probability of *V. thapsus* to survive and flower (Gross, 1981). When treated with soil biota from the native range, plants showed decreased performance due to their loss of defense against specialist soil‐borne pathogens that may be absent in the exotic range. In their review, Bossdorf et al. (2005) found only few studies providing a full test of the EICA hypothesis by addressing growth and defense in the same species, however mostly implying studies that consider herbivore effects. Several of these studies found increased growth or decreased resistance in introduced populations. To our knowledge, there is only one study explicitly testing the EICA hypothesis of plants and soil biota from the native and introduced range of a successful invader. Shelby et al. (2016) compared performance of three *Trifolium* species native to Europe that were introduced to New Zealand but found no differences in competitive ability of introduced and native provenances when grown with soil biota from either the native or exotic range.

A further explanation for the interaction pattern encountered could be specialized mutualists that increase nutrient availability and therefore the competitive ability of plants when grown with soil biota communities of the same origin. Yang et al. (2013) compared plant–soil feedbacks using soil and genotypes from the native and exotic range of *Triadica sebifera* and displayed significant responses for biomass and survival in the native but not in the exotic range. They suggested evolutionary variation in plant–fungi interactions to influence range expansion of exotic plants. In contrast, for *Solidago gigantea*, Maron et al. (2015) found no differentiation among soil genotypes in plant–soil feedbacks. However, we will have to take into account that there is more conditionality behind plant–soil feedbacks, in particular, when addressing biogeographic settings as we did (Maron et al., 2015). In natural settings, the strength of plant–soil feedbacks implies associations with the abundance of individuals, variation among plant genotypes, and variation in the effects of soil from geographically disparate sites. Maron et al. (2015) highlight that different plant genotypes vary in their response to pathogenic agents in soil and that the strength of feedback generated in soils from different locations. This emphasizes the need to consider appropriate sample sizes for such tests in order to account for such differences in native–exotic range comparisons. While we were able to implement five populations of origin by status, there is evidence that accounting for genetic heterogeneity within species would require a higher number of populations to be tested as representatives by status groups to avoid effects of nonrandom geographic sampling (Colautti & Lau, 2015; Rosche et al., 2019).

## 5 CONCLUSION

We tested the role of soil‐borne fungi and bacteria for the successful range expansion of *V. thapsus* in New Zealand by comparing the effects of soil microbes from the native and exotic range on the performance of native and exotic plant populations. Although we found no reliable evidence for soil microbes to be crucial for successful exotic range expansion, we showed that plants originating from different populations differ genetically in many traits and that soil‐borne fungi and bacteria significantly affect different functional plant traits. This emphasizes the particular role soil biota might play in deciding on failure or success of plant invasions (Dickie et al., 2010). Furthermore, it seems that *V. thapsus* populations from the native and exotic range differ in their interaction with different soil microbes and thus suggest plant–soil microbe coevolution. Given that many studies found populations from the native and the exotic range of an exotic plant species to differ genetically (Bossdorf et al., 2005; Colautti & Lau, 2015) and that soil biota are not equally distributed around the globe (Rout & Callaway, 2012), this finding may change our perspective on plant invasions. Thus, to understand the role of soil biota in the invasion context, more is needed to address the effect of soil biota from the home and exotic range on native or exotic populations. Future studies comparing the effect of soil biota from the home and exotic range should broaden such experimental approaches to multispecies testing in multiple exotic ranges in order to increase the generalizability of the findings and prevent missing important parts of the puzzle that might be necessary to get the whole picture.

## CONFLICT OF INTEREST

None declared.

## AUTHOR CONTRIBUTIONS

**Julia Dieskau:** Data curation (lead); formal analysis (equal); investigation (lead); writing–original draft (equal); writing–review and editing (equal). **Helge Bruelheide:** Formal analysis (equal); resources (supporting); supervision (supporting); writing–original draft (supporting); writing–review and editing (supporting). **Jessica Gutknecht:** Methodology (equal); writing–original draft (supporting); writing–review and editing (supporting). **Alexandra Erfmeier:** Conceptualization (lead); formal analysis (equal); investigation (supporting); supervision (lead); writing–original draft (equal); writing–review and editing (equal).

## Data Availability

The data used for this study were deposited on the Dryad Digital Repository (https://doi:10.5061/dryad.4xgxd257b), following the data accessibility guidelines of Ecology and Evolution.
